# Evolution of sexual dimorphism of wing shape in the *Drosophila melanogaster *subgroup

**DOI:** 10.1186/1471-2148-9-110

**Published:** 2009-05-20

**Authors:** Nelly A Gidaszewski, Michel Baylac, Christian Peter Klingenberg

**Affiliations:** 1 Faculty of Life Sciences, University of Manchester, Michael Smith Building, Oxford Road, Manchester M13 9PT, UK; 2 Department of Animal and Plant Sciences, University of Sheffield, Alfred Denny Building, Western Bank, Sheffield S10 2TN, UK; 3 Museum National d'Histoire Naturelle, Laboratoire d'Entomologie, 45 rue Buffon, 75005 Paris, France

## Abstract

**Background:**

Sexual dimorphism of body size has been the subject of numerous studies, but few have examined sexual shape dimorphism (SShD) and its evolution. Allometry, the shape change associated with size variation, has been suggested to be a main component of SShD. Yet little is known about the relative importance of the allometric and non-allometric components for the evolution of SShD.

**Results:**

We investigated sexual dimorphism in wing shape in the nine species of the *Drosophila melanogaster *subgroup. We used geometric morphometrics to characterise wing shape and found significant SShD in all nine species. The amount of shape difference and the diversity of the shape changes evolved across the group. However, mapping the divergence of SShD onto the phylogeny of the *Drosophila melanogaster *subgroup indicated that there is little phylogenetic signal. Finally, allometry accounted for a substantial part of SShD, but did not explain the bulk of evolutionary divergence in SShD because allometry itself was found to be evolutionarily plastic.

**Conclusion:**

SShD in the *Drosophila *wing can evolve rapidly and therefore shows only weak phylogenetic structure. The variable contribution of allometric and non-allometric components to the evolutionary divergence of SShD and the evolutionary plasticity of allometry suggest that SShD and allometry are influenced by a complex interaction of processes.

## Background

Sexual dimorphism is one of the most striking and widespread sources of phenotypic variation in animals and plants and has therefore attracted considerable interest in evolutionary biology. The evolution of sex dimorphism has been extensively studied, but most studies have concerned dimorphism of size [1-4]. In contrast, sexual shape dimorphism (SShD) has been much less investigated.

Of those studies that considered SShD, most have discussed it as a diagnostic-trait for diverse purposes, such as sex identification or the analysis of ontogeny [5-14]. Only relatively few investigations have specifically considered the evolution of SShD, covering a wide range of study systems including the skull in primates [15-18], body proportions of lizards [19,20], newts [21] or flies [22], the head shape of *Chironomus *larvae [23] and *Lycium *flowers [24]. With few exceptions, these studies used only small numbers of species and no explicit phylogenetic framework for analyzing evolutionary change.

A factor that many studies have identified as playing a particularly important role for SShD is allometry, the relationship between size and shape [25-29]. Particularly in primatology, SShD has often been explained by allometric variation of shape and ontogenetic scaling [6,17,27], which raises the possibility that SShD may evolve primarily as a by-product of the evolution of sexual size dimorphism. To address this question, it is useful to separate an allometric component of SShD, for which size dimorphism accounts, from a non-allometric component of residual SShD from other sources. For instance, several studies in primates found that allometric scaling tends to account for much of SShD, but that non-allometric shape variation also contributes a significant part of SShD [16-18]. Similarly, allometric scaling accounts for most of SShD in some species of lizards [13], but other species show sexual dimorphism in body proportions without sexual size dimorphism [30]. Altogether, these studies suggest that the role of allometry in SShD is more complex than previously assumed and therefore requires systematic investigation.

Here we address this issue by investigating the evolution of SShD and the role of allometry of the wing in the nine species of the *Drosophila melanogaster *subgroup. *Drosophila *is a long-standing model organism in evolutionary biology, and the *melanogaster *subgroup has been particularly well studied [31,32]. The *Drosophila *wing is a model trait in evolutionary studies, which has a remarkable potential to evolve under selection [33,34]. Because the intersections between wing veins provide landmarks that can be located precisely, shape variation is easy to quantify with the landmark-based methods of geometric morphometrics [35] and the *Drosophila *wing has therefore been widely used for morphometric studies [36-40]. SShD in the wing of *Drosophila melanogaster *has been found to be quite constant over latitudinal clines of wing shape on different continents, suggesting that SShD may be evolutionarily constrained [36]. Allometry appears to be one of the evolutionary processes possibly involved in this SShD [36,41], although this question has not been addressed directly. This study uses geometric morphometrics to examine SShD of the wing shape in all nine species of the *melanogaster *subgroup, to test for the presence of a phylogenetic signal in SShD and to study the role of allometry in its evolution.

## Results

### Variation of wing size and shape

**Figure 1:**
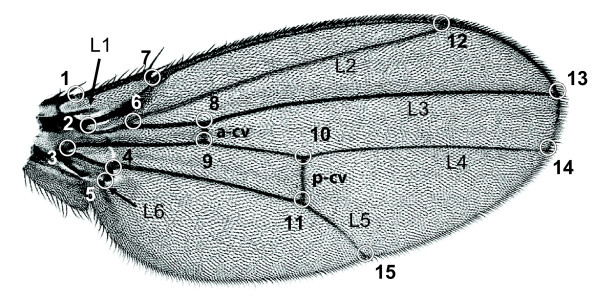
**A *Drosophila *wing and the 15 landmarks used to characterize its shape**. The landmarks are mostly located at intersections between longitudinal veins (L1 to L6), crossveins (a-cv: anterior crossvein, p-cv: posterior crossvein) and the wing margin.

Size and shape of the wings were characterized by a set of 15 landmarks (Figure 1) and analyzed with the methods of geometric morphometrics. To quantify wing size, we used centroid size, which is a measure of the spread of landmarks around their centre of gravity [35]. An analysis of variance indicates that both species and sex have significant effects on centroid size (Table 1). Moreover, the significant species × sex interaction indicates a divergence of sexual size dimorphism among species.

**Table 1:** The effects of sex, species and their interactions on centroid size: Results of the analysis of variance (ANOVA)

Effect	Df	Type III Sum of Squares	Mean squares	*F*	*P*
Species	8	26.581	3.323	298.34	< .0001
Sex	1	14.696	14.696	1319.61	< .0001
Species × sex	8	0.698	0.087	7.84	< .0001

Df: degrees of freedom

Wing shape was extracted from the landmark coordinate data with a generalized Procrustes fit [35]. A multivariate analysis of variance (MANOVA) was used to test the effects of species, sex, size and their interactions on wing shape (Table 2). Species of the *melanogaster *subgroup differ in their wing shapes (significant main effect of species). The MANOVA results also indicate that there is sexual shape dimorphism; however, the marginal significance of the main effect of sex and the highly significant species × sex interaction suggest that sex dimorphism has diverged considerably among species. Allometry contributed to differences in wing shape (significant main effect of centroid size), but there appear to be differences in the allometric patterns among species and sexes (significant interactions of centroid size × sex, centroid size × species and centroid size × species × sex).

**Table 2:** The effects of sex, species and their interaction on wing shape, tested by a multivariate analysis of variance (MANOVA)

Effect	Wilk's lambda	F	Df num	Df den	*P*
Species	0.351	4.36	208	6191.7	< .0001
Sex	0.952	1.54	26	805	0.0411
Csize	0.709	12.69	26	805	< .0001
Species × sex	0.639	1.79	208	6191.7	< .0001
Csize × species	0.379	4.01	208	6191.7	< .0001
Csize × sex	0.951	1.58	26	805	0.0331
Csize × species × sex	0.641	1.77	208	6191.7	< .0001

Df num: degrees of freedom for the numerator; Df den: degrees of freedom for the denominator; Csize: centroid size

### Variation in sexual shape dimorphism

**Figure 2:**
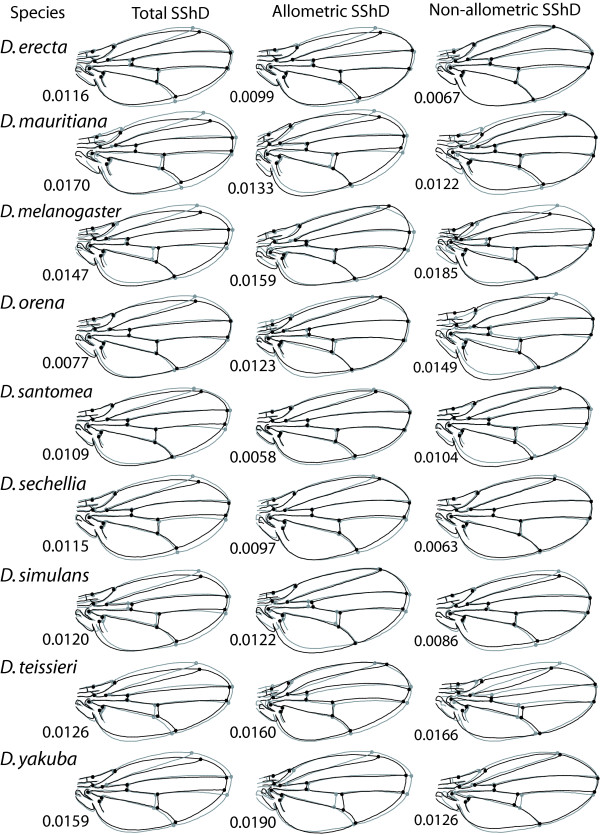
**Shape changes associated with the total, allometric and non-allometric SShD in all nine species**. The shape changes are shown as the difference from the male average shape (grey outlines and hollow circles) to the average shape for females (black outlines and solid circles). All the shape changes are exaggerated 5-fold for better visibility. The total SShD is the raw difference between the male and female average shapes. Allometric SShD is the component due to allometric variation (and therefore in the direction of the allometric vector for the respective species). Finally, non-allometric SShD is the residual component of SShD after subtracting the allometric component from the total SShD. The magnitudes of total shape dimorphism and its allometric and non-allometric components are indicated in units of Procrustes distance.

To quantify the amount of SShD, we computed the Procrustes tangent distance [35] between the mean shapes of the males and females of each species. On the whole, these shape changes were fairly subtle. The magnitudes of total SShD differed among species, with *D. orena *displaying the least shape dimorphism and *D. mauritiana *being the most dimorphic species in the subgroup (Figure 2; numbers next to the diagrams for total SShD).

To visualize the shape changes associated with SShD, we graphed the shape changes from male to female average shapes for all nine species (Figure 2, left). Here we also describe the shape features associated with SShD as changes from the male to female shape. A common feature of SShD in most species is an overall narrowing of the distal part of the wing blade from male to female, particularly in the region between the L3 and L4 veins (landmarks 13 and 14; weaker in *D. erecta*) and to some extent also the distal ends of the L2 and L5 veins (landmarks 12 and 15). This general narrowing was accompanied by various species-specific shape changes from male to female: a posterior shift of landmark 5 (*D. orena*, *D. santomea*, and *D. sechellia*) and a distal shift of the posterior crossvein (*D. mauritiana*, *D. teissieri *and *D. yakuba*), of the anterior crossvein (*D. erecta*) or of both crossveins (*D. melanogaster *and *D. simulans*).

The SShD of different species were distinct from each other (Table 3): pairwise distances between the SShD vectors of different species ranged from 0.0078 (*D. teissieri *versus *D. yakuba*) to 0.0140 (*D. mauritiana *versus *D. melanogaster*). The differences between SShD of different species were therefore of a similar magnitude as the SShD in each species (Figure 2), indicating a considerable diversification. Bootstrap tests [42] against the null hypothesis of equal SShD vectors were all nominally significant (all *P *< 0.05; Table 3) and the false discovery rate [43] is controlled at the 5% level (i.e. it is expected that fewer than 5% of the significant tests are false positives).

**Table 3:** Magnitudes of the differences between the SShD of the nine species

	*D. mauritiana*	*D. melanogaster*	*D. orena*	*D. santomea*	*D. sechellia*	*D. simulans*	*D. teissieri*	*D. yakuba*
*D. erecta*	0.0136 0.0015	0.0135 < 0.0001	0.0113 0.0014	0.0112 < 0.0001	0.0084 0.0053	0.0106 0.0005	0.0118 < 0.0001	0.0120 < 0.0001
*D. mauritiana*		0.0140 0.0005	0.0135 0.0011	0.0133 0.0007	0.0121 0.0041	0.0137 0.0011	0.0132 0.0012	0.0135 0.0011
*D. melanogaster*			0.0127 0.0003	0.0125 < 0.0001	0.0143 < 0.0001	0.0082 0.0254	0.0117 0.0001	0.0124 < 0.0001
*D. orena*				0.0101 0.0101	0.0100 0.0067	0.0095 0.0195	0.0107 0.0041	0.0138 0.0001
*D. santomea*					0.0085 0.0062	0.0120 < 0.0001	0.0127 < 0.0001	0.0105 < 0.0001
*D. sechellia*						0.0113 0.0001	0.0117 < 0.0001	0.0113 < 0.0001
*D. simulans*							0.0104 0.0001	0.0126 < 0.0001
*D. teissieri*								0.0078 0.0022

Tabled values are the magnitude of the difference between the SShD of each pair of species (in units of Procrustes distance, upper value in each table cell) and the *P*-value for the respective bootstrap test of the null hypothesis of no difference in SShD (lower value in each table cell).

**Figure 3:**
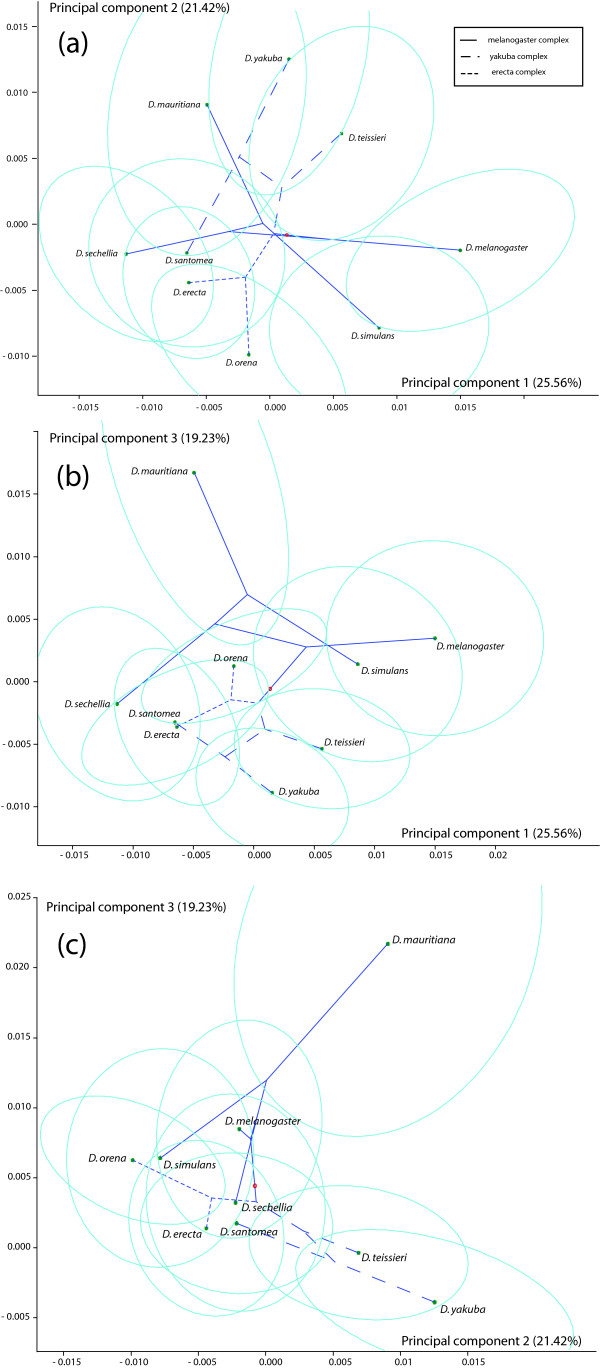
**Divergence of SShD among the species of the *melanogaster *subgroup**. Pairwise plots of the first three principal components (PCs) are displayed. Dots represent the total SShD for each species, associated with 95% confidence ellipses. The phylogenetic tree is projected on each PC plot, with the different complexes of the subgroup (*erecta*, *yakuba *and *melanogaster*) indicated by different types of dashed lines.

The variation of SShD in the *melanogaster *subgroup was displayed graphically by a principal component analysis (Figure 3). None of the principal components (PCs) accounted for a very large proportion of the variation (25.56% for PC1), indicating that the variation of SShD diverged in many directions of the shape space. The confidence ellipses for the SShD of most species were fairly large (Figure 3), which suggests a considerable degree of uncertainty about the SShD for those species. The overlap of the confidence ellipses in the PC plots, despite the significant separation of all species from each other, points to the high-dimensional nature of the variation.

### Phylogenetic signal

**Figure 4:**
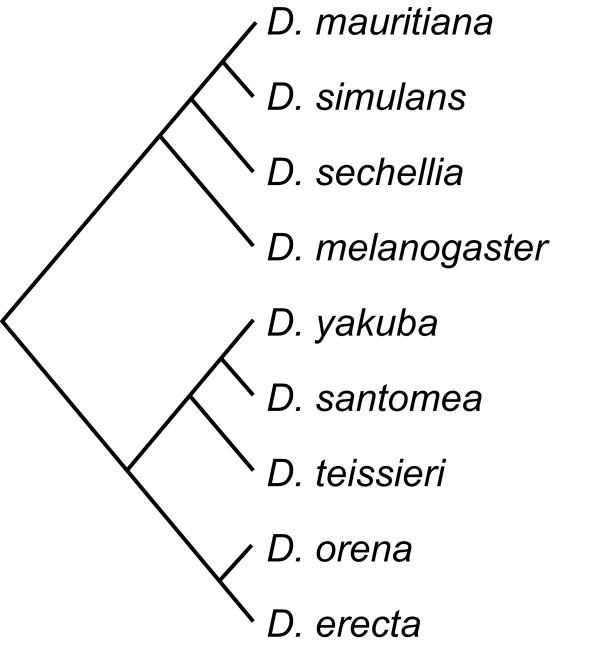
**Phylogenetic tree for the *melanogaster *subgroup**. The tree was compiled from published sources (see Methods).

To reconstruct the evolutionary history of SShD, we mapped the SShD onto a phylogeny of the *melanogaster *subgroup (Figure 4) assembled from published sources [44-46]. The values of SShD corresponding to the internal nodes of the phylogeny were estimated by squared-change parsimony [47,48] and the tree was graphed in the principal component plots [24,49-51] so that reconstructed evolutionary trajectories of SShD can be visualized (Figure 3).

In the resulting graph (Figure 3), many terminal branches of the tree appeared to be fairly long, whereas the internal branches tended to be relatively short. This reflected the fact that some closely related species showed clearly different SShD (e.g. the pairs of sister species *D. simulans *and *D. mauritiana*, *D. santomea *and *D. yakuba*). Moreover, there were many sharp changes of direction in the reconstructed trajectories of evolutionary change and branches appeared to cross in all three PC plots in Figure 3, indicating the presence of homoplasy. Yet, the divergence of SShD also seemed to contain some phylogenetic structure, as the shape dimorphisms of the species belonging to the same complexes were often found in the same areas of the PC plots.

To test for the presence of a phylogenetic signal in SShD, we used a permutation procedure that is a multivariate generalization of the method previously used for scalar traits such as body size [52]. This test simulated the null hypothesis of the complete absence of a phylogenetic signal by randomly permuting SShD vectors among the terminal nodes of the phylogenetic tree and evaluated the resulting tree length for each permutation. The tree length obtained by mapping of the original data onto the phylogenetic tree by weighted squared-change parsimony was 0.000392 (in units of squared Procrustes distance). The permutation procedure resulted in an equal or shorter tree length in nearly half the permutations (proportion 0.48, which corresponds to the *P*-value of the test) and therefore provides no evidence for a phylogenetic signal in SShD.

### Allometry in SShD

**Figure 5:**
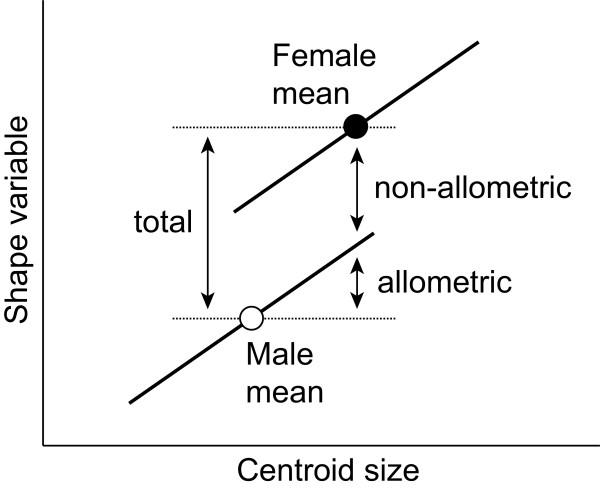
**The decomposition of total sexual shape dimorphism in allometric and non-allometric components**. Allometric and non-allometric components of SShD are quantified by a multivariate regression of shape on centroid size (for simplicity, the regression is displayed in just two dimensions, i.e. with a single shape variable). Allometric regression lines in females and males are assumed to be parallel. The allometric component of SShD is the shape change predicted by the size difference between sexes, and the non-allometric component is the difference between this and the total SShD.

To assess the role of allometry for SShD, we decomposed the total SShD for each species into allometric and non-allometric components (Figure 5). We used multivariate regression of shape on centroid size [53] as the general approach to analyze allometry. To obtain a common estimate for the allometry in both sexes, we ran a pooled within-sex regression in each species. The allometric component of SShD is the part predicted by the sexual size dimorphism (and is thus in the same direction in the shape tangent space as the regression vector), whereas the non-allometric component is the difference between the total SShD and the allometric component (Figure 5).

Because this approach assumes that the allometric regression vectors in males and females are parallel, we conducted a bootstrap test [42] of the null hypothesis that the regression vectors for males and females are the same. There was a significant and substantial difference between regression vectors for males and females in *D. melanogaster *(sum of squared differences 0.023; *P *= 0.022) and smaller differences in *D. santomea *(0.0045; *P *= 0.0094), *D. orena *(0.00096; *P *= 0.037), *D. teissieri *(0.0050; *P *= 0.023). The differences in the other species were small (0.0011 – 0060) and not statistically significant (*P*-values 0.12 – 0.55). Therefore, the separation of allometric and non-allometric components of SShD for *D. melanogaster *is likely to be unreliable, and should be interpreted with some caution in the remaining species as well.

The magnitudes of the allometric and non-allometric components of SShD tended not to add up to the magnitude of the total SShD (Figure 2), as would be expected if all are in the same direction of shape tangent space. Instead, the magnitudes of the allometric and non-allometric components were variable and often of a similar magnitude as the total SShD. In some species, the magnitudes of both components exceeded that of the total SShD (*D. melanogaster*, *D. orena*, *D. teissieri*), but these were among the species where the allometric regressions differed significantly between the sexes, so that this result may be related to poor estimates of a common allometric regression. In all species, however, the magnitudes of the total SShD and its components suggested that the direction of total SShD and the allometric component (and therefore also the non-allometric component) differ considerably.

This expectation was confirmed by inspection of the shape changes associated with SShD and its components (Figure 2). The allometric component of SShD was associated with a wide range of shape changes, which differed considerably from those of total SShD in most species. Allometric SShD tended to consist of a slight expansion of the proximal part of the wing and various changes in the distal part of the wing, which often included a distal shift of landmark 12. In contrast, non-allometric SShD did not include a distal shift of landmark 12 or only a slight one. Non-allometric SShD also showed variety of other shape changes, but there were no other shape features that were shared by most species and there was no apparent relation to the relatedness of species.

## Discussion

This study has yielded three main results concerning the evolution of sex dimorphism in *Drosophila *wing shape. First, we have shown that SShD has diverged among the nine species of the *Drosophila melanogaster *subgroup. Second, the evolution of SShD is little constrained by the phylogenetic structure of the subgroup. Third, allometry is an important component of sexual shape dimorphism in the wing but is not its main evolutionary driver. Here we present the implications of our results for the evolution of the *Drosophila *wing and sexual shape dimorphism.

Our study found substantial evolution of SShD in the *Drosophila melanogaster *subgroup. The MANOVA of shape revealed a clearly significant sex × species interaction (Table 2), which indicates that the effect of sex depends on the species. Similarly, most of the pairwise comparisons of SShD showed highly significant differences between species (Table 3). With a range of 0.0078 to 0.014 (in units of Procrustes distance, Table 3), the magnitudes of pairwise differences between the SShD of different species are therefore of a similar to the magnitudes of SShD, which ranged from 0.0077 to 0.017 (same units, Figure 2). This result is in marked contrast with the finding of Gilchrist et al. [36], who found SShD to be remarkably conserved across latitudinal clines of wing shape that had evolved separately on three continents. Those authors attributed the relative evolutionary conservatism of SShD to constraints by high genetic correlations between male and female wing traits [54], which would be expected to impede the evolution of differences between the sexes. As Gilchrist et al. [36] pointed out, such constraints by genetic correlations can be broken over longer-term evolution. Our results suggest that this may have happened in the evolution of the *melanogaster *subgroup. At the evolutionary scale of the entire clade, rather than a single species, SShD appears to be a trait that readily evolves.

The shape changes involved in SShD consist of the same features that are widely observed in variation within or between populations of *Drosophila *[36,38,40], such as proximal or distal shifts of the crossveins along the longitudinal veins or variation in the positions at which the L2 and L5 veins meet the wing margin. For SShD in different *Drosophila *species, shape features like these seem to be combined in various ways. The PCA of SShD variation among species suggested that variation is distributed over many dimensions rather than being concentrated primarily in one or a few dimensions (the PC1 accounted for 25.6% of the variation and the subsequent eigenvalues decreased gradually). Moreover, the scatter of SShD in shape space is irregular with no dominant patterns or strongly preferred directions (Figure 3). This suggests that evolutionary divergence may flexibly combine a set of wing shape features to make up the SShD for each species.

This impression of flexibility is further reinforced by the observation that there was little phylogenetic structure in the divergence of SShD. There appeared to be no association between relatedness of species and the difference of their SShD (Table 3). The mapping of SShD onto the phylogeny by squared-change parsimony indicated that changes tended to be small for internal branches and large for terminal branches (Figure 3), suggesting that much of the evolutionary divergence of SShD is separately derived for each species. This overall impression was confirmed by the permutation test that yielded a clearly non-significant result, consistent with the null hypothesis of the lack of any phylogenetic structure in SShD.

Allometry has been generally recognized to be a key factor for SShD [6,13,16-18,27] and previous studies have demonstrated allometry in *Drosophila *wings [36,41,55]. Our study confirms that allometry is an important component in the evolution of SShD in the *melanogaster *subgroup; yet, the results also suggest that allometry is not a stringent constraint on the evolution of SShD. In all nine species, both the allometric and non-allometric components of SShD are fairly large (at least half the magnitude of the total SShD, Figure 2). On the one hand, this indicates that allometry can account for a considerable part of SShD, but on the other hand, it also shows that the directions of the total SShD and the allometric regression vector differed considerably from each other and that a substantial part of SShD therefore cannot be explained by allometry. Clearly, factors other than allometry are contributing substantially to SShD in the *melanogaster *subgroup. This is in agreement with studies in other organisms [13,16-18] that also have found non-allometric components of SShD.

Not only is there a substantial contribution by non-allometric SShD, which suggests that allometry is not a severe constraint on the evolution of SShD, but the allometries themselves seem to be variable. The shape features associated with allometry differ from species to species (Figure 2) and the bootstrap test indicated that for some species even the allometries of the two sexes are not the same. This suggests that allometry itself can evolve, which may further erode its role as an evolutionary constraint.

Overall, SShD appears to be a trait that can readily respond to selection or may evolve by drift. The absence of a detectable phylogenetic signal may be the consequence of such selection that has overridden phylogenetic structure in SShD or, alternatively, it may be the result of evolution by random drift (e.g., in association with speciation and founder events). It is plausible that both sexual selection and natural selection affect the evolution of SShD. For instance, the convergent evolution of an unusual sexual dimorphism of body shape in two distantly related species of flies appears to be due to sexual selection from male-male interactions [22], whereas dimorphism of body proportions in lizards has been related to ecological factors [19,20]. In *Drosophila*, the two most likely selective pressures influencing the evolution of SShD in the wing are flight and the production of courtship song. Because females fly to find oviposition sites whereas males search mating opportunities, flight requirements and optimal wing shapes may differ between sexes and result in different regimes of natural selection. Such a difference has been reported for a midge, where different flight behaviour in males and females is associated with sexual shape dimorphism in the wings [56]. Sexual selection may affect SShD in *Drosophila *wings because males use the wings for producing courtship songs with species-specific characteristics [57]. Experimental alteration of sexual selection can produce changes in courtship song [58], which may be related to changes in wing shape. Because the species of the *melanogaster *subgroup live in a range of different environments [59,60], flight behaviour and mating systems differ between species and are likely to yield divergent regimes of natural and sexual selection that may affect SShD in the wings. Because little is known about natural or sexual selection on wing shape, any hypotheses about their effects on SShD must remain very tenuous until more information is available.

## Conclusion

This study characterised SShD in all nine species of the *Drosophila melanogaster *subgroup. We showed a significant divergence of SShD in this clade. There was no detectable phylogenetic signal in the differences, and SShD has both allometric and non-allometric components. Overall, there was little evidence for evolutionary constraints and our results suggested that SShD can readily evolve in response to selection. Particularly because little is known about the natural and sexual selection on wing shape in *Drosophila*, the mechanisms involved in the evolution of SShD are far from being understood.

## Methods

### Samples and measurements

We used flies from long-term laboratory populations of each of the species known to belong to the *Drosophila melanogaster *subgroup: *D. erecta *(49 females; 51 males), *D. mauritiana *(47 females; 43 males), *D. melanogaster *(49 females; 53 males), *D. orena *(49 females; 50 males), *D. santomea *(50 females; 50 males), *D. sechellia *(50 females; 34 males), *D. simulans *(47 females; 37 males), *D. teissieri *(52 females; 53 males), *D. yakuba *(52 females; 52 males). The right wing of each fly was mounted on a microscope slide in a 70% lactic acid/30% ethanol solution and photographed in standard conditions with camera mounted on a Leica microscope.

Wing shape was assessed with 15 landmarks located at vein intersections with other veins and margins (Figure 1). The landmarks were digitised from the images with ImageJ [61] and a plug-in specifically written for this purpose.

### Morphometric and statistical analyses

To quantify wing size, centroid size [35] was computed from the raw coordinates of the landmarks. A generalised Procrustes superimposition [35] was performed on the landmark coordinates to extract the information on wing shape (Procrustes coordinates). All morphometric and statistical analyses were performed using SAS 9.01 software [62], including routines for the Procrustes fit as well as the permutation and bootstrap procedures.

Because sex dimorphism is computed from the mean sizes or shapes of the sexes within each species (unlike, e.g., studies of fluctuating asymmetry [63]) and because our sample sizes are relatively large, measurement error is not a serious concern. Moreover, the methods like those used to acquire coordinate data produce measurement errors that are much smaller than the variation among individuals [39], so that the effect of measurement error on the estimates of sex dimorphism or their statistical variability is negligible.

The effects of species and sex on centroid size were tested with an ANOVA (type III sums of squares). The effects of species, sex and centroid size on shape were tested with a MANOVA (type III sums of squares and cross-products).

SShD for each species was computed as the mean vector for female shape minus the mean vector for male shape. Because male and female samples are independent within each species, covariance matrices for SShD were computed as the sum of the covariance matrices for the mean shapes of the males and females.

To compare SShD between pairs of species, we used a bootstrap test [42] against the null hypothesis that the species share the same SShD. To simulate this null hypothesis, the data for each pair of species were first modified so that they had the same SShD. Bootstrap samples were then repeatedly drawn from the modified data, SShD was computed in both species, and the magnitude of the difference in SShD was then compared to the value obtained from the original data. For each pairwise combination of species, 10,000 rounds of bootstrap resampling were used.

The principal component analysis of SShD was based on the covariance matrix of the SShD vectors for the nine species, and therefore maintains the metric of the Procrustes tangent distances [35]. The 95% confidence ellipses [64] for PC scores were computed from the covariance matrices for SShD of the respective species.

### Phylogenetic signal in SShD

The topology of the phylogenetic tree of the *Drosophila melanogaster *subgroup was assembled as a consensus from the highly supported phylogenetic information based on the entire genomes of five species of the *melanogaster *subgroup [46] together with single-sequence phylogenies of the entire subgroup [44,45] and information from several other studies focussing on specific species complexes [65-69]. Points of disagreement concerned the rooting of the tree, for which the whole-genome analyses [46] provide decisive support, and the sequence of splits between *D. simulans*, *D. sechellia *and *D. mauritiana*, where several sources of evidence favour an earlier divergence of *D. sechellia *[69-71]. Branch lengths for the resulting consensus tree (Figure 4) were compiled from published estimates of divergence times [66,72-75].

To reconstruct the SShD corresponding to the internal nodes of the phylogeny, we used squared-change parsimony, weighted by branch lengths [47,48]. To visualize evolutionary trajectories, we computed the PC scores corresponding to the reconstructed SShD values for the internal nodes of the phylogeny and included them in the PC plots of Figure 3 [49-51].

To test for a phylogenetic signal in SShD against the null hypothesis of the absence of any phylogenetic signal, we used a permutation procedure that is a multivariate extension of a method developed by Laurin [52]. The test uses a permutation approach [76] to simulate the null hypothesis that there is no phylogenetic signal. The nine observed SShD vectors were reassigned to the terminal nodes of the phylogenetic tree in all 362,880 possible permutations of the original order. For each permutation, the new values were mapped onto the phylogeny by weighted squared-change parsimony and the tree length (the sum of the squared Procrustes distances between ancestral and descendant SShD for all branches of the tree) was computed. If the phylogenetic signal in the data is strong, it is expected that random permutation of SShD will usually result in a greater tree length than that of the original data. Therefore, the test computes the *P*-value as the proportion of permutations that result in a tree length that is equal to or less than the one observed for the original data (more detail in Klingenberg and Gidaszewski, unpublished).

### Allometric and non-allometric components of SShD

Total SShD was decomposed into allometric and non-allometric components of variation in each species (Figure 5). We characterized allometry with a multivariate regression of shape (Procrustes coordinates) on centroid size [53]. To obtain a single allometric regression for each species, we used pooled within-sex regression of shape on centroid size.

To test the assumption that the allometric regression is the same in both sexes of each species, we conducted bootstrap tests [42] with the null hypothesis of a common allometric regression between sexes. To simulate this null hypothesis, separate regressions of shape on centroid size were conducted in each sex and the residuals from these regressions were computed. A pooled within-sex regression of shape on centroid size was then used to obtain a common regression vector. The predicted values from this common regression were added to the residuals of the two separate within-sex regressions to generate a modified dataset for which the null hypothesis of a common allometry in both sexes holds true, but which otherwise corresponds to the original samples. Bootstrap samples for males and females were randomly drawn, with replacement, from the modified dataset and the multivariate regressions of shape on centroid size were computed. To quantify the difference between the regression vectors, the sum of squared differences between corresponding elements was computed, and this value was compared to the difference obtained from the allometric regressions with the original data. The bootstrap resampling procedure was repeated 10,000 times for each species and the achieved significance level was computed as the proportion of the rounds of bootstrap resampling for which this difference was equal to or greater than the difference obtained for the original data.

The allometric component of SShD was computed as the vector of coefficients of allometric regression times the difference of centroid size between males and females (Figure 5). We computed the non-allometric component of SShD as the total SShD minus the allometric SShD. The magnitudes of total SShD and the allometric and non-allometric components are the lengths of the corresponding vectors, expressed in units of Procrustes distance.

## Authors' contributions

The project was initiated by CPK and MB. MB arranged for samples of several species. NAG prepared the wings, collected the data and performed the analyses. CPK supervised the project and programmed ImageJ and SAS routines used in the analyses. NAG and CPK wrote the paper.
